# Electro‐Mechanical Uncoupling of K_V_7.1 Voltage Sensor and Pore by 1,4‐Benzodiazepines Is Modulated by Decoration of Position 1

**DOI:** 10.1002/ardp.70305

**Published:** 2026-07-12

**Authors:** Florian Roßner, Thomas Jepps, Bo Hjorth Bentzen, Nathalie Strutz‐Seebohm, Bernhard Wünsch, Guiscard Seebohm

**Affiliations:** ^1^ GRK 2515, Chemical Biology of Ion Channels (Chembion) Universität Münster Münster Germany; ^2^ Institute of Pharmaceutical and Medicinal Chemistry University of Münster Münster Germany; ^3^ Department of Biomedical Sciences University of Copenhagen Copenhagen Denmark; ^4^ Department of Cardiovascular Medicine, Institute for Genetics of Heart Diseases (IfGH) University Hospital Münster Münster Germany

**Keywords:** agonist, gating, K_V_7.1, modifier, (R)‐L3 analogues, structure

## Abstract

The voltage‐gated potassium channel K_V_7.1 (KCNQ1) is essential for cardiac repolarization. Loss‐of‐function mutations prolong the action potential and cause long QT syndrome 1, predisposing to malignant arrhythmias. Pharmacological activators of K_V_7.1 are therefore of therapeutic interest. Among them, the 1,4‐benzodiazepine derivative (R)‐L3 is a potent activator that not only increases current amplitude but also slows activation and deactivation kinetics and abolishes inactivation by uncoupling the voltage sensor from the pore. To explore the structure–activity relationships (SAR) of (R)‐L3, we synthesized and functionally characterized a series of novel 1,4‐benzodiazepine derivatives and examined their effects on K_V_7.1 gating. Human K_V_7.1 channels were heterologously expressed in *Xenopus laevis* oocytes. Two‐electrode voltage clamp recordings were performed to assess current amplitude and kinetic parameters of activation, deactivation, and inactivation. 1,4‐Benzodiazepines modified at 1‐position reproduced the canonical effects of (R)‐L3, including increased current amplitude and suppression of inactivation to varying degrees. Some derivatives displayed completely altered profiles: Modulation of activation, altered (de‐)activation kinetics or exerting attenuated effects on inactivation could be uncoupled. These differences suggest that modifications of the 1,4‐benzodiazepine scaffold at 1‐position shift the interaction between pore binding and voltage sensor–pore uncoupling to isolate kinetic effects. Our data demonstrates that (R)‐L3 analogues can differentially modulate K_V_7.1 gating. By identifying structural determinants of efficacy, this study provides a framework for rational design of next‐generation K_V_7.1 activators. Such compounds may serve as pharmacological tools for dissecting electromechanical coupling in K_V_7.1 and hold promise as candidates for antiarrhythmic therapy in long QT syndrome.

## Graphical abstract

Loss‐of‐function mutations of the voltage‐gated potassium channel K_V_7.1 (KCNQ1) can lead to malignant arrhythmia. The effects of five 1,4‐benzodiazepines modified at the lactam on K_V_7.1 gating were thoroughly analysed by two‐electrode voltage clamp experiments. These analogues could differently modulate kinetic parameters of activation, deactivation and inactivation of the K_V_7.1 channel.

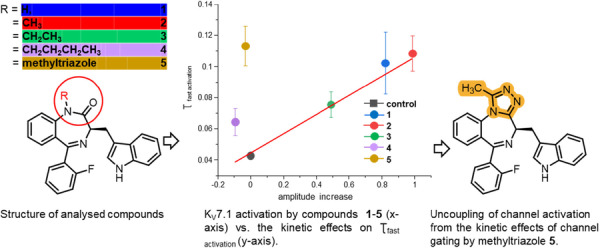

## 1 Introduction

Voltage‐gated potassium (K_V_) channels are fundamental regulators of excitability in excitable tissues such as the heart, brain, (smooth) muscle and pancreas. Within this family, K_V_7.1 (encoded by *KCNQ1*) is of particular importance in the heart, where it assembles with the auxiliary subunit KCNE1 to form the slow delayed rectifier potassium current I_Ks_ that contributes critically to cardiac repolarization during the plateau phase of the action potential during β–adrenergic stimulation [1, 2, 3]. The α‐helical KCNE1 transmembrane region dynamically interacts with the voltage sensor–pore modules of KCNQ1 in a state‐dependent manner [4, 5, 6, 7, 8, 9]. Loss‐of‐function mutations in KCNQ1 or KCNE1 reduce I_Ks_, prolong action potential duration (APD), and cause congenital long QT syndromes (LQT1 and LQT5), which predispose patients to ventricular arrhythmia and sudden cardiac death [3, 10, 11]. Consistently, due to its dominant K^+^‐conductance during β–adrenergic mediated stress reactions, loss‐of‐function in I_Ks_ represents the main trigger for LQT1 and LQT5 caused arrhythmias [12, 13]. Pharmacological augmentation of K_V_7.1 activity has therefore emerged as a potential therapeutic strategy to restore repolarization reserve and prevent arrhythmogenesis [13, 14, 15, 16].

Several small‐molecule activators of K_V_7.1 have been described, including fenamates, zinc pyrithione, Rottlerin, ML277, and the benzodiazepine derivative (R)‐L3 [7, 14, 17, 18, 19, 20, 21, 22, 23]. ML277 is a potent and selective activator that increases current amplitude and slows deactivation without affecting other K_V_7 family members or major cardiac ion channels [20]. The 1,4‐benzodiazepine (R)‐L3 exhibits a complex pharmacological profile. Originally denominated as L‐364,373, (R)‐L3 robustly activates K_V_7.1, increases current amplitude, and markedly alters channel kinetics [14, 19, 20, 22]. Specifically, (R)‐L3 not only slows activation and deactivation but also suppresses inactivation, effectively locking channels in conductive states [19, 20, 22]. In wild‐type K_V_7.1 channels, inactivation is reflected in a ‘hook’ observed in tail currents upon repolarization [24, 25, 26], which disappears in the presence of (R)‐L3 [19]. This suggests that the compound prevents the transition of channels into sub‐conductive or inactivated states.

Mechanistic dissection of (R)‐L3 action revealed that it engages both pore and voltage‐sensing domains (VSD) [20]. Complementary work by Schreiber et al. (2022) [22] extended these findings, demonstrating that (R)‐L3 interacts with residues in the lower S4 helix (e.g., M238, L236, L239) as well as the S4–S5 linker residue W248 [22]. These interactions appear to uncouple voltage sensor movement from pore opening and closing. (R)‐L3 prevents dynamic highly localized interaction of the S4 (M238) with S5 (I271), thereby preventing inactivation [22]. Molecular dynamics simulations further indicated that (R)‐L3 binding stabilizes the activated‐open state of the channel [22]. The functional impact of these mechanisms is significant. By prolonging open‐state occupancy and abolishing inactivation, (R)‐L3 substantially increases repolarizing current. In cardiac myocytes, this translates into shortening of APD and suppression of early afterdepolarizations (EADs), a phenomenon implicated in disease settings of impaired repolarization [14]. Thus, (R)‐L3 represents both a pharmacological tool for studying K_V_7.1 gating and a lead structure for potential therapeutic development. Despite this recent progress, important gaps remain. In particular, the structure–activity relationships (SAR) of (R)‐L3 analogues are poorly defined. It is not known how specific modifications of the benzodiazepine scaffold alter current potentiation, gating kinetics, and the extent of inactivation suppression. Clarifying these relationships is essential to rationally design next‐generation K_V_7.1 activators with optimized pharmacological profiles.

The present study aims to address this gap. We report the synthesis and functional characterization of a series of novel (R)‐L3 analogues. Using two‐electrode voltage clamp recordings and kinetic analyses, we investigate their effects on K_V_7.1 activation, deactivation, and inactivation. By comparing their actions to those of (R)‐L3, we delineate structural determinants of efficacy and elucidate how chemical modifications translate into distinct gating outcomes. Our findings provide new insights into the mechanisms of K_V_7.1 modulation and establish a framework for the rational development of targeted activators for antiarrhythmic therapy.

## 2 Results and Discussion

**Figure 1:**
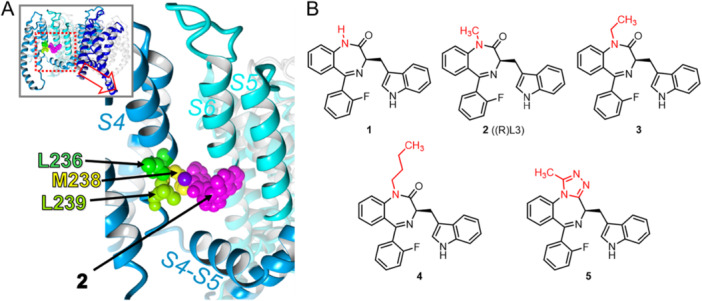
Rationale and chemical structures of **(R)‐L3 (2)** and its analogues **1** and **3**‐**5**. (A) Docking model of (R)‐L3 in K_V_7.1 [22]. Chemical modification strategy at 1‐position of the 1,4‐benzodiazepine scaffold. Residues in S4 that interact with the methyl group of (R)‐L3 (position coloured purple, (R)‐L3 coloured in magenta) *in silico* are shown in space fill. (B) Chemical structures of (R)‐L3 (**2**) and its analogues **1** and **3**‐**5**. Modifications in 1‐position are coloured in red.

To investigate the structural requirements for K_V_7.1 activation, various alkyl substituents were introduced in 1‐position of the 1,4‐benzodiazepine scaffold resulting in the 1‐alkylated derivatives **2**–**4**, including (R)‐L3 (**2**). Additionally, the secondary lactam (**1**) was synthesized. In compound **5**, the methylated lactam of (R)‐L3 was bioisosterically replaced by a methyl triazole moiety (Figure 1). This set of analogues enabled us to explore how specific structural modifications of the benzodiazepine ring system influence the pharmacological modulation of K_V_7.1 stimulation and gating.

**Figure 2:**
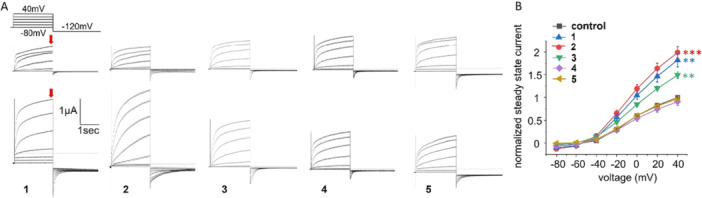
Representative TEVC K_V_7.1 current traces and current–voltage relation. (A) Example current traces recorded from *Xenopus laevis* oocytes expressing K_V_7.1 in control (upper traces) and compound‐treated conditions (lower traces, *c* = 10 µM). (B) Current amplitudes at the end of depolarizing pulses (indicated by red arrows) were determined and normalized mean results are plotted versus voltage of the depolarizing pulse. 1,4‐Benzodiazepines **1**–**3** exert clear activation of K_V_7.1 channel currents. Student's *t*‐test on currents at 40 mV revealed significant current increase upon application of compound **1**: *p* = 0.0013 (indicated by **), compound **2**: *p* = 0.0003 (indicated by ***) and compound **3**: *p* = 0.0011 (indicated by **); *n* = 8‐11.

Two‐electrode voltage clamp recordings of K_V_7.1 expressed in *Xenopus laevis* oocytes revealed that the tested compounds **1, 2** and **3** (*c* = 10 µM) increased macroscopic K_V_7.1 currents (Figure 2A). The steady‐state current–voltage relationship (Figure 2B) demonstrates that some 1,4‐benzodiazepine analogues significantly increased current density (**1**–**3**) whereas other compounds were ineffective (**4, 5**). The functional analyses show a pronounced increase in current amplitude only for compounds without a substituent in 1‐position (**1**) or with a small methyl (**2**) or ethyl moiety (**3**) in 1‐postion compared to control. Thus, only short alkyl residues are tolerated for macroscopic channel activation, which follows the rule the shorter the alkyl chain the more effective in stimulating K_V_7.1 channels. However, distinct compound‐dependent kinetic effects on activation and deactivation (tail currents) were observed.

**Figure 3:**
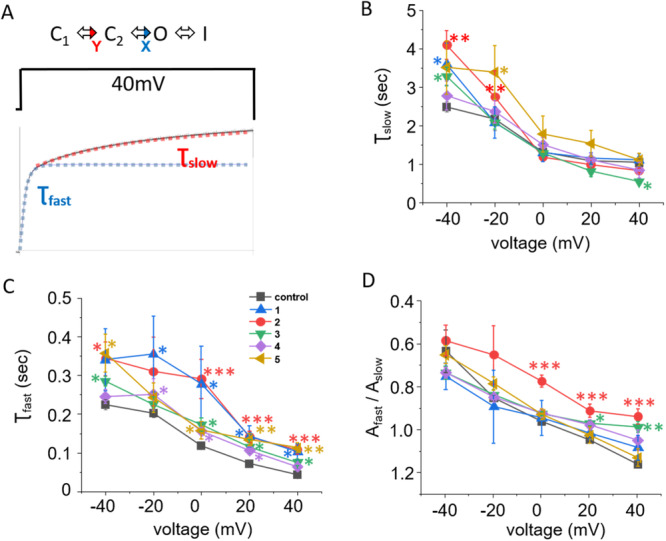
Activation kinetics of K_V_7.1 currents. (A) K_V_7.1 activates with two distinct kinetic components (biexponential activation kinetics). An example trace is shown at 40 mV. An exponential fit describing the fast (blue) and the slow (red) components is shown in dotted lines. In a simple linear gating scheme these components are accounted for by deep (C_1_) and intermediate (C_2_) closed states. (B, C) Voltage dependence of the slow (Ʈ_slow_) and fast (Ʈ_fast_) activation time constants. Time constants of the slow and fast components were plotted versus voltage. (D) Ratio of amplitudes (A_fast_/A_slow_) as a function of voltage. Student's *t*‐test on values at a respective voltage revealed significant changes upon application. Significant changes are indicated by *p ≤ 0.05; **p ≤ 0.01 and ***p ≤ 0.001 in the colour code of the respective compound; *n* = 3–14.

Activation kinetics were fitted with a double‐exponential function, yielding fast (Ʈ_fast_) and slow (Ʈ_slow_) activating components [15, 24]. All active compounds slowed both components compared to control, but to different extents (Figure 3B,C). Methyl triazole **5** exhibited the strongest effect, markedly prolonging Ʈ_slow_ (Figure 3B), while the secondary lactam (**1**) and the butyl derivative **4** caused only moderate changes. The ethyl derivative **3** exerts only mild increase in slow activation. The ratio of amplitudes (A_fast_/A_slow_) increased with depolarization, suggesting a relative decrease of the slow activation component. Representative traces (Figure 2A) and statistical analyses (Figure 3D) illustrate the compound‐induced slowing of current onset. Together, these results suggest that 1,4‐benzodiazepines promote occupancy of the open state, with (R)‐L3 (**2**) exerting the most prominent kinetic activation modulation.

**Figure 4:**
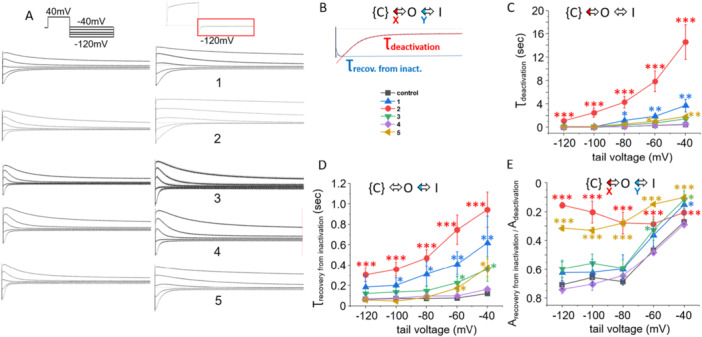
Deactivation kinetics and recovery from inactivation. (A) Pulse protocol to elicit currents. Channels were activated by depolarization to +40 mV (2 s) and stepping back to voltages of −120 to −40 mV in 20 mV increments (4.5 s). Representative tail currents are shown from before application (left traces) and after application of the indicated compounds (right traces). The tail currents are scaled to similar control size to allow for better vision on the compounds effect. Tail currents show two distinct kinetic components (biexponential kinetics). (B) An example trace is shown at −120 mV. An exponential fit describing the recovery from inactivation (blue) and the deactivation (red) components is shown in coloured lines. In a simple linear gating scheme, these components are accounted for with an inactivated state (I) and (O) open state(s). (C) Voltage dependence of deactivation time constants (Ʈ_deactivation_) versus voltage is represented. (D) Voltage dependence of recovery from inactivation (Ʈ_recovery_) plotted versus voltage is shown. (E) Ratio of recovery from inactivation to deactivation amplitudes. The relative amplitude of the inactivation component versus the amplitude of the deactivating component plotted versus voltage is shown. Student's *t*‐test on values at a respective voltage revealed significant changes upon application, Significant changes are indicated by *p ≤ 0.05; **p ≤ 0.01 and ***p ≤ 0.001 in the colour code of the respective compound; *n* = 3–14.

Deactivation and recovery from inactivation were analysed using tail current analyses [15, 24, 25]. Analysis of tail currents revealed that (R)‐L3 (**2**) and to a smaller degree derivatives **1** and **5** slowed deactivation, as reflected by increased Ʈ_deactivation_ across hyperpolarized potentials (Figure 4C). In parallel, recovery from inactivation was modified, with increased Ʈ_recovery from inactivation_ values compared to control (Figure 4D). Notably, the ratio of amplitudes of component recovery from inactivation to component deactivation was strongly shifted in the presence of compounds **2** and **5** (Figure 4E), consistent with stabilization of the open state and facilitation of channel reopening.

Channel availabilities at 40 mV [27] were determined and resulted in control: 0.52 ± 0.04 (*n* = 9), compound **1**: 0.54 ± 0.06 (*n* = 4), compound **2**: 0.86 ± 0.02 (*n* = 6), compound **3**: 0.56 ± 0.03 (*n* = 5), compound **4**: 0.43 ± 0.06 (*n* = 6) and compound **5**: 0.70 ± 0.03 (*n* = 6). Compound **2** and compound **5** highly significantly increase apparent channel availability (compound **2**: *p* = 3.5 × 10^−8^ and compound **5**: *p* = 8.6 × 10^−7^). Representative traces (Figure 4A) highlight the pronounced slowing of deactivation and slowed recovery kinetics.

**Figure 5:**
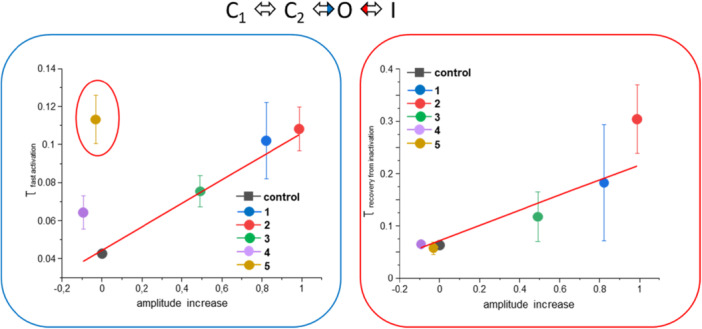
Correlation of kinetic parameters with activating compound effects. Scatter plots of kinetic parameters (Ʈ_fast activation_ at 40 mV (blue) and Ʈ_recovery from inactivation_ at −120 mV (red)) plotted versus activation (amplitude increase) of respective compound at 10 µM. Linear regression shows significant correlations of these parameters, whereas **5** does not follow the correlation of Ʈ_fast activation_ to activation.

**Figure 6:**
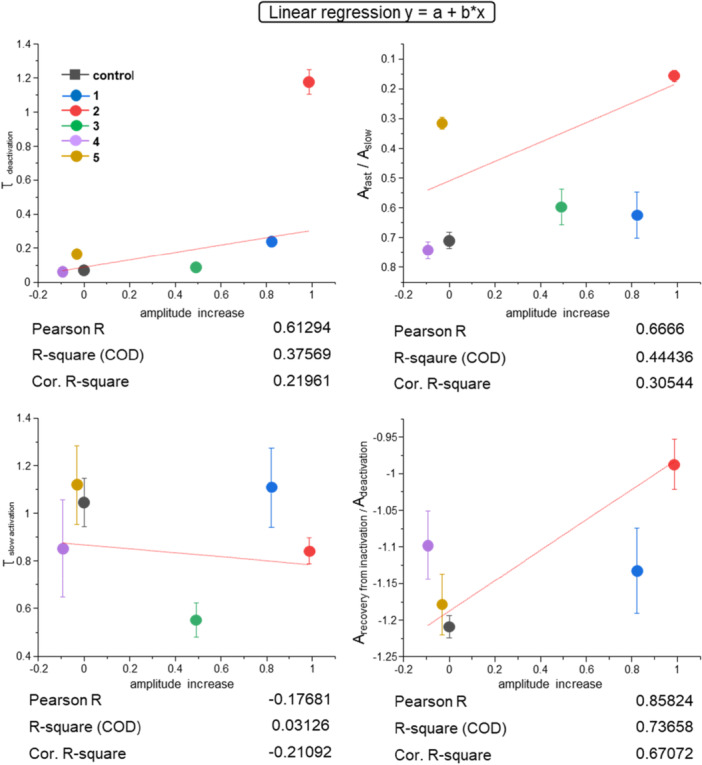
Lack of correlation of kinetic parameters with other compound effects. Scatter plots of kinetic parameters plotted versus activation (amplitude increase) of respective compound at 10 µM concentration. Linear regression shows no significant correlations of these parameters to stimulation. Values of linear regressions shown underneath the respective graph.

To obtain further mechanistic insights, linear regression analyses of kinetic parameters with stimulating compound effects were conducted (Figure 5). Scatter plots of kinetic parameters (Ʈ_fast activation_ and Ʈ_recovery from inactivation_) versus amplitude increase of compound shown and linear regressions shows significant correlations of these parameters, whereas triazole‐annotated benzodiazepine **5** does not follow the correlation of Ʈ_fast activation_ to activation (Pearson R: 0.73336; R^2^ (COD): 0.53781; Cor. R^2^: 0.42226). Thus, linear regression without **5** resulted in even higher correlation (Pearson R: 0.91347; R^2^ (COD): 0.83443; Cor. R^2^: 077924). Activating effects correlate mildly with the time course of recovery from inactivation (Pearson R: 0.83122; R^2^ (COD): 0.69092; Cor. R^2^: 0.61365). Interestingly, in this case **5** follows the trend, which indicates that the inactivation and activating effects of the compounds can be separated and that the effect on fast activation might be mechanistically coupled to activation and voltage sensor movement [20, 22]. Linear regression analyses of the other determined parameters showed only weak or no correlations between the kinetic parameters Ʈ_slow activation_ and Ʈ_deactivation_ and the amplitudes relationships that were detected (Figure 6).

## 3 Conclusion

In this study, we investigated the effects of novel 1,4‐benzodiazepine derivatives on the gating properties of the K_V_7.1 channel. Our results demonstrate that chemical modifications of the benzodiazepine scaffold can differentially modulate current amplitude, activation and deactivation kinetics, as well as the extent of inactivation suppression. These findings extend our previous work on the parent 1,4‐benzodiazepine (R)‐L3 (**2**) and shed new light on the molecular basis of K_V_7.1 activation [19, 22]. A central observation is that some analogues reproduced the canonical features of (R)‐L3, namely increased current amplitude and reduced inactivation [14, 19, 20, 22]. However, other analogues displayed a shift in their relative effects, for example, by primarily influencing activation kinetics while only modestly affecting inactivation. This divergence underscores the importance of distinct structural determinants within the (R)‐L3 ‐ 1,4‐benzodiazepine pharmacophore for controlling the balance between pore‐domain and voltage sensor–domain interactions.

Earlier work established that ML277 and (R)‐L3 both bind within a hydrophobic pocket in the S5–S6 region of the K_V_7.1 pore [19, 20, 22]. The 1,4‐benzodiazepine (R)‐L3 not only stabilizes the open pore conformation but also uncouples voltage sensor movements, thereby abolishing inactivation [19, 20, 22]. Our analogue data are consistent with the notion that substitutions on the benzodiazepine scaffold alter the compound's capacity to engage residues in the selectivity filter, pore domain and lower S4 segment (e.g., M238, R237, H240) and the S4–S5 linker (W248) [19, 20, 22]. Thus, fine‐tuning the N1 substituents on 1,4‐benzodiazepines may selectively enhance or weaken its ability to interfere with voltage sensor–pore coupling to shift the channels towards open/active channels and increase macroscopic current amplitudes. Annulation of a triazole ring at (R)‐L3 led to compound **5**. This compound incorporates a novel combination not observed in 1‐alkylated 1,4‐benzodiazepines. The triazolobenzodiazepine **5** showed little activation but can abolish inactivation as observed by the recovery from inactivation indicated by the ‘hook in the tail’ [15, 24, 25, 26]. The ability to reduce or abolish K_V_7.1 inactivation can be useful as a tool compound and may have potential therapeutic implications. In native cardiac myocytes, inactivation of homomeric K_V_7.1 is attenuated by co‐assembly with KCNE1 [1, 2]. However, there exists a subset of Long QT1‐associated mutations like K_V_7.1(L273F) that introduces inactivation to the K_V_7.1/KCNE1 channel complex, thereby functionally causing a loss‐of‐function of the I_Ks_ current. Such long QT1 mutations might benefit from a compound counteracting inactivation [26]. Nevertheless, (R)‐L3 reproduces some of the effects of KCNE1 by preventing inactivation and slowing deactivation [14, 19]. Certain analogues like **1** in our study appeared to mimic or even enhance this property more selectively, suggesting that structural modifications could yield compounds with improved pharmacological specificity. Such agents might enhance I_Ks_ function even in the presence of KCNE1, a limitation of both ML277 and (R)‐L3 [20].

Importantly, not all analogues showed identical efficacy, and in some cases, modifications reduced activity almost completely. This highlights the delicate balance between maintaining favourable binding interactions and avoiding steric hindrances within the pore–VSD interface. Using a combination of state and structural modelling we have suggested that (R)‐L3 favours binding to open states of K_V_7.1 [22]. Analogue‐dependent differences in activity may therefore reflect changes in binding affinity to specific channel states, which could be tested by future experiments, molecular dynamics simulations and structural determinations with bound compounds in cryoEM.

From a scientific perspective, we present compounds with differing activating and kinetic effects on K_V_7.1 channels that may represent novel tool compounds for ion channel research. However, analyses of the effects at different concentrations of the compound would be helpful in rounding out our understanding of how they work. From a translational perspective, the identification of analogues that potently suppress inactivation while exerting moderate effects on activation kinetics may be particularly promising. Excessive slowing of activation could delay I_Ks_ onset and be counterproductive in the setting of rapid heart rates, whereas selective inhibition of inactivation would increase repolarization reserve without impairing physiological channel dynamics. Future *in vivo* studies to determine how these changes in channel gating translate to physiological effects seem warranted. However, preliminary additional *in vitro* studies on hERG‐channels to evaluate the safety and selectivity of these compounds would be necessary.

Taken together, our findings demonstrate that (R)‐L3 analogues can be tailored to differentially modulate K_V_7.1 gating. By mapping their effects onto known binding determinants in the S4–S6 interface, we provide a framework for rational drug design aimed at developing next‐generation K_V_7.1 activators/modulators. These compounds may serve as valuable tools for dissecting the mechanisms of voltage sensor–pore coupling and hold therapeutic potential for arrhythmias associated with I_Ks_ dysfunction.

## 4 Experimental Part

### 4.1 Synthesis of Novel (R)‐L3 Analogues

The design, synthesis and structural proof of novel (R)‐L3 analogues modified in 1‐position has been described in ‘Structure‐activity relationship study on ligands activating the voltage‐gated potassium channel K_V_7.1’.

### 4.2 Molecular Biology and cRNA Preparation

Human *KCNQ1* (K_V_7.1 encoding) cDNA was subcloned into the oocyte expression vector psGEM. Plasmids were linearized with NheI and transcribed *in vitro* using T7 RNA polymerase using the mMESSAGE mMACHINE kit (Thermo Fisher), as described previously [22]. The integrity of the RNA was confirmed by agarose gel electrophoresis and concentration was determined photometrically. For experiments with homomeric K_V_7.1 channels, 5 ng of cRNA were injected per oocyte.

### 4.3 Oocyte Handling and Two‐Electrode Voltage Clamp (TEVC)

Stage V–VI oocytes were obtained from Ecocyte Bioscience (Castrop Rauxel, Germany). Oocytes were stored at 18°C in ND96 supplemented with 1.8 mM CaCl_2_, 50 mg/L gentamicin, and 2.5 mM sodium pyruvate. Microinjections of KCNQ1 cRNA were performed using a Nanoliter 2000 injector (World Precision Instruments, Friedberg, Germany). Injected oocytes were incubated for 2–3 days at 18°C before recordings. Two‐electrode voltage clamp (TEVC) recordings were used to measure currents. Currents were recorded at room temperature (22°C–24°C) using a Turbo Tec 10CD amplifier (NPI Electronic GmbH, Tamm, Germany) in standard ND96 bath solution (in mM: NaCl 96, KCl 2, CaCl_2_ 1.8, MgCl_2_ 1, HEPES 5, pH 7.4). Microelectrodes were filled with 3 M KCl and had resistances of 0.5–1.5 MΩ. Signals were digitized using a National Instruments NI USB‐6221 interface (National Instruments, Austin, TX, USA) and GePulse software (Michael Pusch, Genova, Italy). Leak subtraction was not applied. Depolarizing protocols consisted of 2‐s test pulses from −80 to +40 mV in 20 mV increments from a holding potential of −80 mV, followed by repolarization to −120 mV to elicit tail currents. Kinetic analysis of activation and inactivation—Activation time courses were fitted with bi‐exponential function:

$$I_{\left(t\right)}=A_{0}+A_{\text{fast}}\times \text{exp}-t/{Ʈ}_{\text{fast}}+A_{\text{slow}}\times \text{exp}-t/{Ʈ}_{\text{slow}},$$

where Ʈ_fast_/A_fast_ and Ʈ_slow_/A_slow_ represent the fast and slow activation components.

Inactivation was quantified from the reduction of current amplitude during depolarizing pulses and by analyzing recovery from inactivation during tail currents as described earlier [15]. In brief, deactivation and recovery from inactivation were studied by repolarization steps (−120 to −40 in 20 mV increments) following a 2‐s depolarization to +40 mV. Tail current decays/increases were fitted to bi‐exponential functions. Recovery from inactivation was further quantified from the ‘hook’ in tail currents, that is, an initial rising phase reflecting channels returning from the inactivated to the open state before deactivation. The fast component was assigned to recovery from inactivation, and the slow component to channel deactivation. Recovery kinetics were described by time constants derived from exponential fits of this rising phase. Deactivation and recovery from inactivation time courses were fitted with bi‐exponential function:

$$I_{\left(t\right)}=A_{0}+A_{\text{recovery from inactivation}}\times \text{exp}-t/{Ʈ}_{\text{recovery from inactivation}}+A_{\text{deactivation}}\times \text{exp}-t/{Ʈ}_{\text{deactivation}},$$

where A_recovery from inactivation_/exp − t/Ʈ_recovery from inactivation_ and A_deactivation_/exp − t/Ʈ_deactivation_ represent the fast recovery from inactivation and slow deactivation components.

The fraction of available channels was quantified by the ratio of the initial tail current (x) to an extrapolated current from a single exponential fit starting after the hook (y), as previously described by Yu et al. [27].

Linear regressions were done to the formula y = a + b × x using Origin Pro (vers. 2020b; OriginLab, Northampton, MA, USA). All analyses of electrophysiological data were performed with Ana software (Michael Pusch, Genova, Italy) and Origin Pro. Data are presented as mean ± SEM. Statistical significance was determined using one‐way ANOVA or unpaired Student's *t*‐test, with *p* < 0.05 considered significant.

### 4.4 Molecular Model Visualization

Molecular modelling and visualization of K_V_7.1‐(R)‐L3 model were performed in YASARA 20 as previously described [22] and the potentially (R)‐L3 interacting residues in S4 are shown.

## Conflicts of Interest

The authors declare no conflicts of interest.

## Supporting information


Supporting File

## Data Availability

The data that support the findings of this study are available on request from the corresponding author. The data are not publicly available due to privacy or ethical restrictions.
