# Supplementary material for: Electro‐Mechanical Uncoupling of KV7.1 Voltage Sensor and Pore by 1,4‐Benzodiazepines Is Modulated by Decoration of Position 1
Source: Arch Pharm (Weinheim). 2026 Jul 12;359(7):e70305. doi: 10.1002/ardp.70305 (PMC13358183; doi:10.1002/ardp.70305)
Supplement: Supplementary file 1 — Supporting File [file ARDP-359-e70305-s001.docx]

**InChi Codes**

**Electro-Mechanical Uncoupling of Kv7.1 voltage sensor and pore by 1,4-Benzodiazepines is modulated by decoration of position 1**

Florian Roßner, Thomas Jepps, Bo Hjorth Bentzen, Nathalie Strutz-Seebohm, Bernhard Wünsch^*^, and Guiscard Seebohm ^*^

| **compd.** | **InChICode** |
| --- | --- |
| **1** | InChI=1S/C24H18FN3O/c25-19-10-4-1-8-17(19)23-18-9-3-6-12-21(18)28-24(29)22(27-23)13-15-14-26-20-11-5-2-7-16(15)20/h1-12,14,22,26H,13H2,(H,28,29)/t22-/m1/s1 |
| **2** | InChI=1S/C25H20FN3O/c1-29-23-13-7-4-10-19(23)24(18-9-2-5-11-20(18)26)28-22(25(29)30)14-16-15-27-21-12-6-3-8-17(16)21/h2-13,15,22,27H,14H2,1H3/t22-/m1/s1 |
| **3** | InChI=1S/C26H22FN3O/c1-2-30-24-14-8-5-11-20(24)25(19-10-3-6-12-21(19)27)29-23(26(30)31)15-17-16-28-22-13-7-4-9-18(17)22/h3-14,16,23,28H,2,15H2,1H3/t23-/m1/s1 |
| **4** | InChI=1S/C28H26FN3O/c1-2-3-16-32-26-15-9-6-12-22(26)27(21-11-4-7-13-23(21)29)31-25(28(32)33)17-19-18-30-24-14-8-5-10-20(19)24/h4-15,18,25,30H,2-3,16-17H2,1H3/t25-/m1/s1 |
| **5** | InChI=1S/C26H20FN5/c1-16-30-31-26-23(14-17-15-28-22-12-6-3-8-18(17)22)29-25(19-9-2-5-11-21(19)27)20-10-4-7-13-24(20)32(16)26/h2-13,15,23,28H,14H2,1H3/t23-/m1/s1 |
